# Novel biomarkers and endoscopic techniques for diagnosing pancreaticobiliary malignancy

**DOI:** 10.12688/f1000research.11371.1

**Published:** 2017-09-05

**Authors:** Margaret G Keane, Amar Shah, Stephen P Pereira, Deepak Joshi

**Affiliations:** 1 Institute of Liver Studies, King's College Hospital, London, UK; 2 UCL Institute for Liver and Digestive Health, Royal Free Campus, London, UK

**Keywords:** Pancreaticobiliary malignancy, Endoscopic retrograde, cholangiopancreatography, CA 19-9, pancreatic, biliary

## Abstract

The UK incidence of pancreatic ductal adenocarcinoma is 9 per 100,000 population, and biliary tract cancer occurs at a rate of 1–2 per 100,000. The incidence of both cancers is increasing annually and these tumours continue to be diagnosed late and at an advanced stage, limiting options for curative treatment. Population-based screening programmes do not exist for these cancers, and diagnosis currently is dependent on symptom recognition, but often symptoms are not present until the disease is advanced. Recently, a number of promising blood and urine biomarkers have been described for pancreaticobiliary malignancy and are summarised in this review. Novel endoscopic techniques such as single-operator cholangioscopy and confocal endomicroscopy have been used in some centres to enhance standard endoscopic diagnostic techniques and are also evaluated in this review.

## Introduction

In the UK, pancreatic ductal adenocarcinoma (PDAC) is the 10 ^th^ commonest cancer and has an incidence of 9 per 100,000 population ^1^, and biliary tract cancer (BTC) (including intra- and extra-hepatic cholangiocarcinoma and gallbladder cancer) has an incidence of 1–2 cases per 100,000 population ^2^. Long-term survival is poor; 5-year survival is less than 4% for both tumours ^3, 4^. Often these tumours are diagnosed late, when patients have advanced disease and curative surgical resection is no longer possible.

Globally the highest incidence of PDAC is seen in Northern Europe and North America ^5^, where the rates are 3 to 4 times higher than in tropical countries ^6^. Overall incidence is increasing ^5^, and as most tumours are sporadic, this rising incidence is attributed to differences in lifestyles and exposure to environmental risk factors ^7^, such as smoking ^8– 15^, diabetes mellitus, chronic pancreatitis ^1, 15, 16^ and obesity ^17^.

In BTC, the variations in incidence seen globally are even more pronounced; and the highest incidence is in northeastern Thailand (96 per 100,000 men) ^18^, which has a population with high levels of chronic typhoid and infestation of liver fluke ( *Clonorchis sinensis* and *Opisthorchis viverinni*) ^18^. Other BTC risk factors seen in all populations include older age ^18^, primary sclerosing cholangitis ^19^, intraductal stones and rare biliary cystic diseases ^20^. Inflammatory bowel disease, chronic viral hepatitis, cirrhosis, smoking, diabetes, obesity and excess alcohol consumption may also increase the risk of BTC ^20– 22^.

Despite improved diagnostic techniques, detecting pancreaticobiliary malignancy remains a significant clinical challenge. A common presentation of these tumours is a biliary stricture with or without a mass lesion. The differential of an indeterminate biliary stricture is broad, and often the associated symptoms and radiological findings overlap between benign and malignant conditions, often making differentiation—particularly between cancer, primary sclerosing cholangitis and IgG4-related disease—impossible without further investigations, typically by endoscopic retrograde cholangiopancreatography (ERCP) or endoscopic ultrasound (EUS) ^23– 25^. However, biliary brush cytology is also an imperfect test, although specificity is high (96–100%), sensitivity for malignancy remains low (9–57%) and in early disease when tumours are small, sensitivities are even lower ^26, 27^. Therefore, patients frequently require multiple procedures to obtain a final diagnosis ^28– 30^.

So there has been growing interest in the development of simple tests to streamline the diagnosis to pancreaticobiliary malignancy and guide appropriate and timely therapy for patients. Identifying better diagnostic tools for PDAC and BTC would also make screening and surveillance possible, particularly in high-risk populations ^4, 8, 31^. This would enable the detection of tumours at an earlier stage when curative resection is possible, leading to substantial improvements in survival ^32^. This review provides an overview of the latest innovations in diagnostic biomarkers and endoscopic techniques for pancreaticobiliary malignancy.

## Methods

We performed a systematic review of the literature by using PubMed, EMBASE and the Cochrane Library. The search was limited to studies published in the English language between January 2013 and March 2017. Medical Subject Headings (MeSH) terms were decided by a consensus of the authors and included “pancreatic cancer” or “cholangiocarcinoma” and “biomarker”. The search was restricted to title, abstract and keywords. Articles that described outcomes for fewer than five patients were excluded. Case reports, abstracts and reviews were excluded. All references were screened for potentially relevant studies not identified in the initial literature search.

The following variables were extracted for each report when available: number of malignant and benign cases, sensitivity, specificity and area under the curve (AUC). One hundred ten articles were included in the final review.

## Biomarkers

### 1. Serum biomarkers and blood tests

Carbohydrate antigen (CA) 19-9 is the most widely used tumour marker in pancreaticobiliary malignancy. Overall sensitivity (78–89%) and specificity (67–87%) are low, and in around 7% of the population who lack the Lewis (a) antigen, CA19-9 will remain negative ^33^. In small tumours, sensitivity decreases further. The marker can also be elevated in a number of other malignant diseases (for example, gastric adenocarcinoma) and benign diseases, particularly those causing jaundice (for example, primary biliary cirrhosis, cholestasis and cholangitis), and in smokers ^34^. In addition, variation has been reported among commercially available assays, which may impact on interpretation ^35^. Therefore, to improve the sensitivity of the marker in current clinical practice, it is always interpreted in the context of cross-sectional imaging findings ^33^.

Other commercially available tumour markers that have a role in diagnosing pancreaticobiliary cancer include carcinoembryonic antigen (CEA) and CA125. CEA is a glycosyl phosphatidyl inositol cell surface-anchored glycoprotein that is involved in cell adhesion. When elevated, it is highly suggestive of colorectal cancer, but it is also increased in about a third of patients with BTC ^36– 38^. CA125 is a protein encoded by the *MUC16* gene and is a large membrane-associated glycoprotein with a single transmembrane domain. When elevated, it is suggestive of ovarian cancer, but it is also increased in about 40–50% of patients with pancreaticobiliary malignancy, particularly when there is peritoneal involvement ^38^.

Owing to the limitations of existing biomarkers, over the last few years several studies have evaluated various combinations of biomarkers to supplement or ultimately replace existing biomarkers. Biomarker panels using combinations of markers, often including CA19-9, have been particularly successful in detecting small tumours and early disease. Validation studies have also shown that these markers can differentiate PDAC from relevant benign conditions and in some cases detect tumours up to 1 year prior to diagnosis with a specificity of 95% and a sensitivity of 68% ^7^ ( Table 1 and Table 2).

**Table 1.** Serum protein biomarkers for biliary tract cancer, 2013–2017.

Author (year)	Biomarker/ Combination (serum)	Biliary tract cancer, number	Benign lesion/ cholangitis, number	Healthy volunteers, number	Sensitivity	Specificity	Area under the curve
Single biomarkers							
Han *et al*. (2013) ^84^	HDGF	83	-	51	66%	88%	0.81
Ruzzenente *et al*. (2014) ^85^	MUC5AC	49	23	16	-	-	0.91
Voigtlander *et al*. (2014) ^86^	Angpt-2	56	111	-	74%	94%	0.85
Lumachi *et al*. (2014) ^87^	CA 19-9	24	25	-	74%	82%	-
Wang *et al*. (2014) ^88^	CA 19-9	78	78	78	72%	96%	-
Lumachi *et al*. (2014) ^87^	CEA	24	25	-	52%	55%	-
Wang *et al*. (2014) ^88^	CEA	78	78	78	11%	97%	-
Wang *et al*. (2014) ^88^	CA 125	78	78	78	45%	96%	-
Lumachi *et al*. (2014) ^87^	CYFRA 21-1	24	25	-	76%	79%	-
Liu *et al*. (2015) ^89^	VEGF-C	31	10	10	71%	80%	0.79
Liu *et al*. (2015) ^89^	VEGF-D	31	10	10	74%	85%	0.84
Huang *et al*. (2015) ^90^	CYFRA 21-1	134	52	-	75%	85%	-
Lumachi *et al*. (2014) ^87^	MMP7	24	25	-	78%	77%	-
Nigam *et al*. (2014) ^91^	Survivin	39 (gallbladder cancer)	30	25	81%	80%	-
Rucksaken *et al*. (2014) ^92^	HSP70	31	12	23	94%	74%	0.92
Rucksaken *et al*. (2014) ^92^	ENO1	31	-	23	81%	78%	0.86
Rucksaken *et al*. (2014) ^92^	RNH1	31	-	23	94%	67%	0.84
Wang *et al*. (2014) ^88^	CA242	78	78	78	64%	99%	-
Ince *et al*. (2014) ^93^	VEGFR3	96	129	-	48%	82%	0.62
Ince *et al*. (2014) ^93^	TAC	96	129	-	61%	60%	0.60
Rucksaken *et al*. (2017) ^94^	ORM2	70	46	20	92%	74%	-
Rose *et al*. (2016) ^95^	CEACAM6	41	42	-	87.5%	69%	0.74
Jiao *et al*. (2014) ^96^	Nucleosides	202 (gallbladder cancer)	203	205	91%	96%	-
Biomarker combinations							
Lumachi *et al*. (2014) ^87^	CEA + CA19-9 + CYFRA 21-1 + MMP7	24	25	-	92%	96%	

**Table 2.** Serum protein biomarkers for pancreatic cancer, 2012–2017.

Author (year)	Biomarker/ Combination (serum)	PDAC, number	Benign controls, number	Healthy volunteers, number	Sensitivity	Specificity	Area under the curve
Single biomarkers							
Sogawa *et al*. (2016) ^97^	C4BPA	52	20	40	67%	95%	0.860
Rychlikova *et al*. (2016) ^98^	Osteopontin	64	71	48	-	-	-
Lin *et al*. (2016) ^99^	APOA-I	78	-	36	96%	72.2%	0.880
Lin *et al*. (2016) ^99^	TF	78	-	36	75%	72.8%	0.760
Guo *et al*. (2016) ^100^	Dysbindin	250	80	150	81.9%	84.7%	0.849
Han *et al*. (2015) ^101^	Dickkopf-1	140	-	92	89.3%	79.3%	0.919
Qu *et al*. (2015) ^102^	DCLK1	74	74	-	-	-	0.740
Dong *et al*. (2015) ^103^	Survivin	80	-	80	-	-	-
Gebauer *et al*. (2014) ^104^	EpCAM	66	43	104	66.7%	77.5%	-
Wang *et al*. (2014) ^105^	MIC-1	807	165	500	65.8%	96.4%	0.935
Kendrick *et al*. (2014) ^106^	IGFBP2	84	40	84	22%	95%	0.655
Kendrick *et al*. (2014) ^106^	MSLN	84	40	84	17%	95%	0.668
Kang *et al*. (2014) ^107^	COL6A3	44	46	30	-	-	0.975
Willumsen *et al*. (2013) ^108^	C1M	15	-	33	-	-	0.830
Willumsen *et al*. (2013) ^108^	C3M	15	-	33	-	-	0.880
Willumsen *et al*. (2013) ^108^	C4M	15	-	33	-	-	0.940
Willumsen *et al*. (2013) ^108^	C4M12a1	15	-	33	-	-	0.890
Falco *et al*. (2013) ^109^	BAG3	52	-	44	75%	75%	0.770
Falco *et al*. (2013) ^109^	BAG3	52	17 (chronic pancreatitis)	-	81%	77%	0.810
Chen *et al*. (2013) ^110^	TTR	40	-	40	91%	47%	0.730
Gold *et al*. (2013) ^111^	PAM4	298	-	79	76%	96%	-
Gold *et al*. (2013) ^111^	PAM4	298	120	-	-	-	0.890
Poruk *et al*. (2013) ^112^	OPN	86	48	86	-	-	0.720
Poruk *et al*. (2013) ^112^	TIMP-1	86	48	86	-	-	0.770
Lee *et al.* (2014) ^113^	CA 19-9	41	12	44	80.4%	70%	0.833
Lee *et al.* (2014) ^113^	Human complement factor B (CFB)	41	12	44	73.1%	97.9%	0.958
Mixed cohorts							
Ince *et al*. (2014) ^93^	CEA	96 (41 PDAC +25 BTC)	129	-	42.7%	89.9%	0.713
Ince *et al*. (2014) ^93^	CA19-9	96 (41 PDAC +25 BTC)	129	-	49%	84.5%	0.701
Ince *et al*. (2014) ^93^	VEGFR3	96 (41 PDAC +25 BTC)	129	-	48.4%	82.9%	0.622
Ince *et al*. (2014) ^93^	Total antioxidant capacity	96 (41 PDAC +25 BTC)	129	-	61.1%	60.5%	0.602
Abdel-Razik *et al*. (2016) ^114^	IGF-1	47 (25 PDAC + 18 BTC)	62	-	62%	51%	0.605
Abdel-Razik *et al*. (2016) ^114^	VEGF	47 (25 PDAC + 18 BTC)	62	-	58.3%	57.3%	0.544
Biomarker combinations							
Chen *et al*. (2013) ^110^	TTR + CA19-9	40	-	40	81%	85%	0.910
Lee *et al*. (2014) ^113^	CA19-9 + CFB	41	12	44	90.1%	97.2%	0.986
Sogawa *et al*. (2016) ^97^	C4BPA + CA19-9	52	20	40	86%	80%	0.930
Makawita *et al*. (2013) ^115^	CA19-9 + REG1B	100	-	92	-	-	0.880
Makawita *et al*. (2013) ^115^	CA19-9 + SYCN + REG1B	100	-	92	-	-	0.870
Willumsen *et al*. (2013) ^108^	C1M + C3M + C4M + C4M12a1	15	-	33	-	-	0.990
Shaw *et al*. (2014) ^116^	IL10 + IL6 + PDGF + Ca19-9	84	45 (benign)	-	93%	58%	0.840
Shaw *et al*. (2014) ^116^	IL8 + IL6 + IL-10 + Ca19-9	84	32 (chronic pancreatitis)	-	75%	91%	0.880
Shaw *et al*. (2014) ^116^	IL8 + IL1b + Ca 19-9	127	-	45	94%	100%	0.857
Brand *et al*. (2011) ^117^	Ca-19 + CEA + TIMP-1	173	70	120	71%	89%	-
Capello *et al*. (2017) ^118^	TIMP1 + LRG1 + Ca19-9	73	-	60	0.849%	0.633%	0.949
Capello *et al*. (2017) ^118^	TIMP1 + LRG1 + Ca19-9	73	74	-	0.452%	0.541%	0.890
Chan *et al*. (2014) ^119^	Ca19-9 + Ca125 + LAMC2	139	65	10	82%	74%%	0.870
Makawita *et al*. (2013) ^115^	CA19-9 + REG1B	82	41	92	-	-	0.875
Makawita *et al*. (2013) ^115^	CA19-9 + SYNC + REG1B	82	41	92	-	-	0.873
Makawita *et al*. (2013) ^115^	CA19-9 + AGR2 + REG1B	82	41	92	-	-	0.869

BTC, biliary tract cancer; PDAC, pancreatic ductal adenocarcinoma.

In pancreaticobiliary malignancy and PDAC in particular, metastatic disease occurs at a very early stage in tumour development. This is demonstrated by the fact that patients who underwent resection of small primary tumours (<2 cm) with no clinical evidence of metastatic disease had a 5-year survival after pancreatectomy of less than 18% owing to recurrent metastatic disease ^39^. Tumour development is driven by a series of cumulative genetic abnormalities; therefore, genetic and epigenetic changes have been explored as diagnostic targets in circulating tumour cells, cell-free DNA (cfDNA) and non-coding RNA ( Table 3– Table 5). Owing to the position and composition of pancreaticobiliary tumours, tissue samples are frequently acellular, making diagnostics challenging. Recently, the utility of next-generation sequencing was explored as a technique that allows the detection of low-abundance mutations and abnormalities in small amounts of material ^40^. Changes in the metabalome are also being explored as a potential diagnostic tool in pancreaticobiliary malignancy ^41^.

**Table 3.** Genetic and epigenetic alterations in circulating tumour cells in pancreatic ductal adenocarcinoma and biliary tract cancer, 2013–2017.

Author (year)	Target	Biliary tract cancer, number	Pancreatic ductal adenocarcinoma, number	Benign lesions, number	Healthy volunteers, number	Detected	Sensitivity	Specificity	Area under the curve
Ankeny *et al*. (2016) ^120^	K-ras	-	72	28	-	-	75%	96.4%	0.867
Kulemann *et al*. (2016) ^121^	K-ras	-	21	-	10	80% (stage IIA/IIB) 91% (stage III/IV)	-	-	-
Singh *et al*. (2015) ^122^	ctDNA, K-ras	-	-	-	-	-	65.3%	61.5%	0.6681
Kinugasa *et al*. (2015) ^123^	K-ras	-	141	20	20	-	62.6%	-	-
Takai *et al*. (2015) ^124^	K-ras	-	259	-	-	-	29.2%	-	-
Sausen *et al*. (2015) ^125^	ctDNA	-	77	-	-	-	43%	-	-
Kulemann *et al*. (2015) ^126^	CTC K-ras	-	11	-	9	75% (stage IIb) 71% (stage III)	-	-	-
Zhang *et al*. (2015) ^127^	DAPI ^+^, CD45-, CK ^+^, CEP8 > 2 ^+^	-	22 Validation cohort: 11	6 8	30 10	68.2%	63.6%	94.4%	0.84
Wu *et al*. (2014) ^128^	K-ras	-	36	-	25	-	0	0	-
Bidard *et al*. (2013) ^129^	CK, CD45	-	79	-	-	11%	-	-	-
Bobek *et al*. (2014) ^130^	DAPI, CK, CEA, Vimentin	-	24	-	-	66.7%	-	-	-
Rhim *et al*. (2014) ^131^	DAPI, CD45, CK, PDX-1	-	11	21	19	78%	-	-	-
Iwanicki-Caron *et al*. (2013) ^132^	CTC	-	40	-	-	-	55.5%	100%	-
Sheng *et al*. (2014) ^133^	CTC	-	18	-	-	94.4%	-	-	-
Catebacci *et al*. (2015) ^134^	CTC (in portal venous blood at EUS)	2	14	-	-	100% (pulmonary vein blood) 22.2% (peripheral blood)	-	-	-
Earl *et al*. (2015) ^135^	CTC	-	35	-	-	20%	-	-	-
Cauley *et al*. (2015) ^136^	Circulating epithelial cells	-	105	34	9	49%	-	-	-
Kamande *et al*. (2013) ^137^	DAPI, CD45, CK	-	12	-	-	100%	-	-	-

**Table 4.** Genetic and epigenetic alterations in circulating cell-free DNA pancreatic ductal adenocarcinoma and biliary tract cancer, 2013–2017.

Author (year)	Target	PDAC or BTC	Cancer, number	Benign lesions, number	Healthy volunteers, number	Detected	Sensitivity	Specificity
Takai *et al*. (2016) ^138^	K-ras	PDAC	107 (non- operable)	-	-	59%	-	-
Takai *et al*. (2015) ^124^	cfDNA	PDAC	48			29%		
Hadano *et al*. (2016) ^139^	K-ras	PDAC	105	-	20	31%	-	-
Zill *et al*. (2015) ^140^	K-ras, TP53, APC, FBXW7, SMAD4	PDAC	26	-	-	-	92.3%	100%
Earl *et al*. (2015) ^135^	K-ras	PDAC	31	-	-	26%	-	-
Kinusaga *et al*. (2015) ^123^	G12V, G12D, and G12R in codon 12 of K-ras gene	PDAC	141	20	20	62%	-	-
Sausen *et al*. (2015) ^125^	cfDNA	PDAC	77	-	-	43%	-	-
Wu *et al*. (2014) ^128^	K-ras	PDAC	24	-	25	72%	-	-

BTC, biliary tract cancer; PDAC, pancreatic ductal adenocarcinoma.

**Table 5.** Epigenetics: circulating non-coding RNA and DNA methylation markers in pancreatic ductal adenocarcinoma/biliary tract cancer, 2013–2017.

Author (year)	MicroRNA	Biliary tract cancer, number	Pancreatic ductal adenocarcinoma, number	Benign lesions, number	Healthy volunteers, number	Sensitivity	Specificity	Area under the curve
Circulating non-coding RNA								
Kishimoto *et al*. (2013) ^141^	MiR-21 (↑)	94 94	- -	- 23	50 -	85% 72.3%	100% 91.3%	0.93 0.83
Wang *et al*. (2013) ^142^	miR-27a-3p + CA19-9(↑)	-	129	103	60	85.3%	81.6%	0.886
Kawaguchi *et al*. (2013) ^143^	miR-221 (↑), miR-375 (↓)	-	47	-	30	-	-	0.762
Zhao *et al*. (2013) ^144^	miR-192 (↑)	-	70	-	40	76%	55%	0.63
Carleson *et al*. (2013) ^145^	MiR-375 (↑)	-	48	47	-	-	-	0.72
Que *et al*. (2013) ^146^	miR-17-5p (↑) miR-21 (↑),	-	22	12	8	-	-	0.887 0.897
Schultz *et al*. (2014) ^147^	Index I + CA19-9 Index II + CA19-9	-	409	25	312	85% 85%	88% 86%	0.93 0.92
Silakit *et al*. (2014) ^148^	MiR-192 (↑)	11	-	-	9	74%	72%	0.803
Lin *et al*. (2015) ^149^	MiR-492 (↑) MiR-663a (↑)	-	49	-	27	75% 85%	70% 80%	0.787 0.870
Chen *et al*. (2014) ^150^	miR-182 (↑)	-	109	38	50	64.1%	82.6%	0.775
Wang *et al*. (2015) ^151^	MiR-150 (↑)	15	-	-	15	80%	58%	0.764
Ganepola *et al*. (2015) ^152^	miR-22 (↑), miR-642b (↑) miR-885-5p (↑)	-	11	-	11	91%	91%	0.970
Voigtlander *et al*. (2015) ^153^ (serum)	MiR-1281 (↑) MiR-126 (↑) MiR-26a (↑) MiR-30b (↑) MiR-122 (↑)	31	-	40	-	55% 68% 52% 52% 32%	90% 93% 93% 88% 90%	0.83 0.87 0.78 0.78 0.65
Voigtlander *et al*. (2015) ^153^ (bile)	miR-412 (↑) miR-640 (↑) miR-1537 (↑) miR-3189 (↑)	31	-	53	-	50% 50% 67% 67%	89% 92% 90% 89%	0.81 0.81 0.78 0.80
Abue *et al*. (2015) ^154^	miR-21 (↑), miR-483-3p (↑)	-	32	12	30	-	-	0.790 0.754
Salter *et al*. (2015) ^155^	miR-196a (↑), miR-196b (↑)	-	19	10	10	100%	90%	0.99
Kojima *et al*. (2015) ^156^	miR-6075, miR-4294, miR-6880-5p, miR-6799-5p, miR-125a-3p, miR-4530, miR-6836-3p, miR-4476	98	100	21	150	80.3%	97.6%	0.953
Xu *et al*. (2015) ^157^	miR-486-5p (↑) miR-938 (↑)	-	156	142	65	-	-	0.861 0.693
Madhaven *et al*. (2015) ^158^	PaCIC + miRNA serum-exosome marker panel	-	-	-	-	100%	80%	-
Komatsu *et al*. (2015) ^159^	miR-223 (↑)	-	71	-	67	62%	94.1%	0.834
Alemar *et al*. (2016) ^160^	MiR-21 (↑) MiR-34a (↑)	-	24	-	10	-	-	0.889 0.865
Wu *et al*. (2016) ^161^	MiR-150 (↓)	30	30	28	50	-	-	-
Bernuzzi *et al*. (2016) ^162^	MiR-483-5p(↑) MiR-194(↑)	40	40	70	40	-	-	0.77 0.74
Kim *et al*. (2016) ^163^	mRNA – CDH3 (↑) mRNA –IGF2BP3(↑) mRNA – HOXB7 (↑) mRNA – BIRC5 (↑)	-	21	14	-	57.1% 76.2% 71.4% 76.2%	64.3% 100% 57.1% 64.3%	0.776 0.476 0.898 0.818
Duell *et al*. (2017) ^164^	MiR-10a (↑) MiR-10b (↑) MiR-21-5p (↑) MiR-30c (↑) MiR-155 (↑) MiR-212 (↑)	-	225	-	225	-	-	0.66 0.68 0.64 0.71 0.64 0.64
DNA hypermethylation								
Branchi *et al*. (2016) ^165^	*SHOX2*/ *SEPT9*	20	-	-	100	0.45%	0.99%	0.752

### 2. Bile and biliary brush biomarkers

Patients with an indeterminate stricture on cross-sectional imaging are typically referred for an ERCP and biliary brushing with or without endobilary biopsy to obtain tissue for diagnosis, with or without therapeutic stenting ^28^. Although these techniques do not compromise resection margins in potentially resectable cases, sensitivity remains low (9–57%) and patients frequently have to undergo multiple procedures to obtain a diagnosis ^28– 30^. Bile can be easily obtained at the time of ERCP and, owing to its proximity to the tumour, is a potentially important source of diagnostic biomarkers in these cancers ( Table 6). Unfortunately, owing to the invasiveness of ERCP, the role of these biomarkers is limited to diagnosis rather than screening or surveillance in these tumours.

**Table 6.** Bile and biliary brush biomarkers for pancreatic and biliary tract cancer.

Author (year)	Biomarker	Pancreatic ductal adenocarcinoma, number	Biliary tract cancer, number	Benign lesions, number	Healthy controls, number	Bile or biliary brush	Sensitivity	Specificity	Area under the curve
Single biomarkers									
Dhar *et al*. (2013) ^166^	M2-PK	-	88	79	17	Bile	90.3%	84.3%	-
Navaneethan *et al*. (2015) ^167^	M2-PK	-	-	-	-	Bile	52.9%	94.1%	0.77
Keane (2017) ^168^	MCM5	24	17	47		Biliary brush	55.6%	77.8%	0.79
Danese *et al*. (2014) ^169^	MUC5AC	-	20	20	-	Serum Bile	-	-	0.94 0.99
Farina *et al*. (2014) ^170^	CEAM6	23	6	12	-	Bile	93%	83%	0.92
Budzynska *et al*. (2013) ^171^	NGAL	6	16	18	-	Bile	77%	72%	0.74
Jiao *et al*. (2014) ^96^	Nucleosides		202 (gallbladder cancer)	203	205	Bile	95.3%	96.4%	-
Ince *et al*. (2014) ^93^	CE	41	25	129	-	Bile	57.3%	68.2%	0.516
Ince *et al*. (2014) ^93^	CA 19-9	41	25	129	-	Bile	74.0%	34.1%	0.616
Ince *et al*. (2014) ^93^	VEGFR3	41	25	129	-	Bile	56.2%	79.1%	0.663
Ince *et al*. (2014) ^93^	Total antioxidant capacity	41	25	129	-	Bile	65.6%	50.4%	0.581
Abdel-Razik *et al*. (2016) ^114^	IGF-1	25	18	62	-	Bile	91.4%	89.5%	0.943
Abdel-Razik *et al*. (2016) ^114^	VEGF	25	18	62	-	Bile	90.3%	84.9%	0.915
Kim *et al*. (2016) ^163^	mRNA – CDH3 (↑) mRNA –IGF2BP3(↑) mRNA – HOXB7 (↑) mRNA – BIRC5 (↑)	-	21	14	-	Biliary brush	57.1% 76.2% 71.4% 76.2%	64.3% 100% 57.1% 64.3%	0.776 0.476 0.898 0.818

### 3. Urinary biomarkers

Urine provides a very easy and acceptable source for biomarker analysis. In BTC, a 42-peptide panel (consisting mostly of fragments of interstitial collagens) correctly identified 35 of 42 BTC patients with a sensitivity of 83% and a specificity of 79% ^42^. In PDAC, the three-biomarker panel (LYVE-1, REG1A and TFF1) has been validated in a multi-centre cohort of 371 samples. When comparing PDAC stage I–IIA (resectable disease) with healthy urines, the panel achieved AUCs of 0.97 (95% confidence interval of 0.93–1.00). The performance of the urine biomarker panel in discriminating PDAC stage I–IIA was superior to the performance of serum CA19-9 ( *P*=0.006) ^43^ ( Table 7).

**Table 7.** Summary of urine protein biomarkers for pancreatic and biliary tract cancer, 2013–2017.

Author (year)	Biomarker/ Combination (urine)	Pancreatic ductal adenocarcinoma, number	Biliary tract cancer, number	Benign cancer/ Chronic pancreatitis, number	Healthy volunteers, number	Sensitivity	Specificity	Area under the curve
Single biomarker									
Roy *et al*. (2014) ^172^	MMP2	51	-	-	60	70%	85%	-
Roy *et al*. (2014) ^172^	TIMP-1	51	-	-	60	90%	70%	-
Jiao *et al*. (2014) ^96^	Nucleosides	-	202 (gallbladder cancer)	203	205	89.4%	97.1%	-
Metzger *et al*. (2013) ^42^	Urine Proteomic analysis	-	42	81	-	83%	79%	0.87
Biomarker combinations									
Radon *et al*. (2015) ^43^	LYVE-1 + REG1A + TFF1	192	-	-	87	-	-	0.89

### 4. Symptoms and cancer decision support tools

Recently, pre-diagnostic symptom profiles have been investigated as an alternative way of detecting hepato-pancreato-biliary (HPB) cancers at an early stage ^8, 9, 16, 44^. It is now recognised that the onset of PDAC and BTC is heralded by a collection of gastrointestinal and constitutional symptoms ^45^. Although overlap occurs with other benign and malignant conditions, certain symptoms such as back pain, lethargy and new-onset diabetes have been identified as particularly suggestive of PDAC. Commonly performed blood tests such as liver function tests, glucose and haemoglobin also typically become abnormal in the months preceding diagnosis ^46^. Therefore, cancer decision support tools have been produced from combinations of symptoms and risk factors. In the UK, they have been introduced into general practices in 15 cancer networks to date ^8^, and their utility is currently being audited ^47^. Modification to existing tools to enhance their diagnostic accuracy can be expected in the future.

## Endoscopy

### 1. Endoscopic ultrasonography

If there is a mass lesion on cross-sectional imaging, endoscopic ultrasonography with fine-needle aspiration (EUS-FNA) provides an alternative method for visualising and sampling the extra-hepatic biliary tree, pancreas, gallbladder or peri-hilar lymph nodes. EUS-FNA has a diagnostic accuracy for PDAC of between 65% and 96% ^48, 49^. In BTC, a single-centre study reported a sensitivity of 73%, which was significantly better in distal compared with proximal tumours (81% versus 59% respectively, *P*=0.04) ^50^. Recently, developed fine core biopsy needles appear to have improved diagnostic accuracy over traditional FNA needles, but randomised trials are awaited ^49, 51, 52^. Rapid onsite examination by a cytopathologist is used in some centres, particularly in North America, and has been shown to improve the yield of EUS-FNA in individual centres ^53, 54^ but this trend has not been borne out in recent randomised controlled trials ^55^.

To improve the diagnostic accuracy of EUS, it can also be combined with novel adjuncts such as contrast agents (SonoVue ^®^), transient elastography (TE) or confocal laser endomicroscopy (CLE). TE allows the measurement of the tissue firmness, which tends to be increased in malignant tissue. In a recent single-centre study from the UK, quantitative strain measurements were found to have high sensitivity but low specificity for the detection of PDAC ^56^. The technology to perform the techniques is available on most modern EUS machines and adds little time to the overall procedure time. The technique can be performed equally well by endosonographers with limited experience ^57, 58^ and is particularly advantageous in cases where the diagnosis remains uncertain after standard EUS has been performed ^59^. Contrast-enhanced EUS is performed with agents such as SonoVue ^®^ and allows visualisation of the early arterial phase and late parenchymal phase enhancement of the pancreas. Pancreatic tumours are generally hypovascular compared with the surrounding parenchyma ^60, 61^. Dynamic contrast EUS is a relatively novel method that allows the non-invasive quantification of the tumour perfusion compared with the pancreatic parenchyma by using software that is now built into a number of EUS scanners. The use of this technology is evolving but is expected to be most applicable when predicting tumour response to chemotherapeutic agents, particularly new drugs against vascular angioneogenesis ^62, 63^.

**Figure 1.**
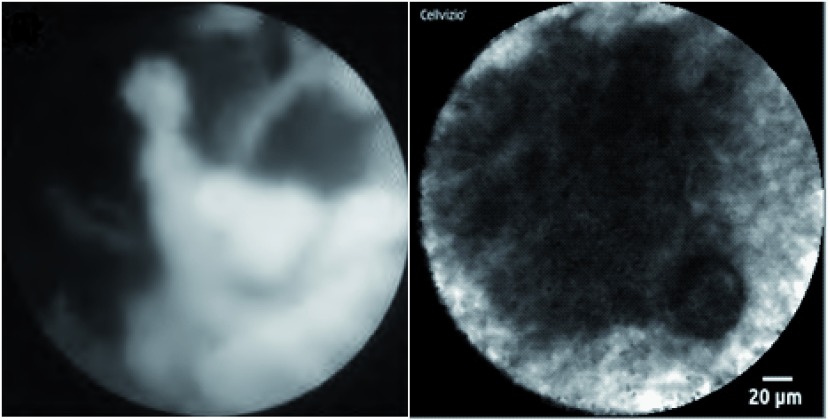
Novel diagnostic adjuncts to ERCP and EUS. ( **a**) Cholangioscopic view of a malignant hilar stricture with visualisation of the ulcerated, friable biliary mucosa via the Spyglass cholangioscope system (Boston Scientific Corp, Massachusetts, USA). ( **b**) Confocal endomicroscopic image of pancreatic cancer, showing characteristic black clumps. Image was obtained using the Cellvizio AQ-Flex® probe which was introduced to the tumour via 19G FNA needle at the time of EUS.

Recently, a needle-based confocal endomicroscope has also been developed which can be passed through a 19G FNA needle to assess indeterminate masses, cysts or lymph nodes. Malignancy in the hepatobilary tract is identified by the presence of irregular vessels, vascular leakage and large dark clumps ( Figure 1) ^64^. In a recent study of 25 patients with indeterminate pancreatic masses referred for EUS-FNA, needle-based CLE was shown to be a safe and feasible technique ^65^.

### 2. Endoscopic retrograde cholangiopancreatography

ERCP is typically undertaken when imaging demonstrates an indeterminate biliary stricture and tissue acquisition is required for cytological or histological assessment. Biliary brush cytology and endobiliary biopsy have a sensitivity for malignancy of 9–57% ^29, 30, 66, 67^. Most HPB tumours exhibit chromosomal aneuploidy ^68^; therefore, in some centres, fluorescence *in situ* hybridisation and digital image analysis are used to assess for the presence of DNA abnormalities in brush cytology ^30, 69^. Although these techniques have been adopted by only a few centres, the presence of polysomy is highly suggestive of BTC ^30, 69^. 

Poor diagnostic accuracy in biliary brush and endobiliary samples has been attributed to their being non-targeted samples obtained with only fluoroscopic guidance ^70^. The single-operator cholangioscopy system (SpyGlass, Boston Scientific Corporation, Natick, MA, USA) introduced in 2006 and now superseded by the SpyGlass DS system enables intrabiliary biopsies under direct vision via small disposable forceps ( Figure 1). In a recent systematic review, the sensitivity and specificity of cholangioscopy-guided biopsies in the diagnosis of malignant biliary strictures were 60.1% and 98.0%, respectively ^71^. Higher sensitivities are observed for intrinsic biliary malignancy compared with extrinsic compressing tumours ^72^. Several techniques have been employed to augment the visualised mucosa during cholangioscopy, including chromendoscopy with methylene blue ^73– 75^, narrow-band imaging ^76, 77^ and autofluorescence ^78^.

During ERCP, a “CholangioFlex” confocal probe (Mauna Kea Technologies, Paris, France) can be placed down the working channel of a cholangioscope or duodenoscope to obtain real-time CLE images, which are akin to standard histology ( Figure 1). If the images obtained from a point on the biliary mucosa contain dark areas, this is highly suggestive of malignancy ^79, 80^. The diagnostic accuracy of probe-based CLE was recently validated in a prospective multi-centre international study with 112 patients (71 with malignant lesions). Tissue sampling alone had a sensitivity, specificity and diagnostic accuracy of 56%, 100% and 72%, respectively. In comparison, ERCP with probe-based CLE had a sensitivity, specificity and diagnostic accuracy of 89%, 71% and 82%, respectively. Diagnostic accuracy increased to 88% when probe-based CLE and tissue sampling results were combined ^81^. CLE is also feasible in the pancreatic duct during pancreaticoscopy but, owing to concerns over pancreatitis, is rarely used. In a case report by Meining *et al*., the presence of a main duct-intraductal papillary mucinous neoplasia was confirmed by clear views of typical finger-like projections ^82^. Intraductal ultrasound in small studies has also been shown to have a diagnostic accuracy of up to 90% ^83^.

## Conclusions

Currently, the most widely used tumour marker in pancreaticobiliary malignancy is CA19-9. However, its use is limited by its elevation in a number of other benign and malignant conditions. Furthermore, it is not produced in approximately 7% of the population who are Lewis antigen–negative and is often undetectable when tumours are small. Over the last few years, a number of very promising biomarker panels have been identified which can detect tumours at an early stage when curative intervention could be possible. These markers are subject to ongoing validation studies but appear likely to be implemented into screening programmes, particularly for high-risk groups, in the near future. Novel endoscopic techniques such as per-oral cholangioscopy and confocal endomicroscopy can enhance the diagnostic accuracy of standard techniques and are increasingly available in large-volume centres worldwide.

## Abbreviations

AUC, area under the curve; BTC, biliary tract cancer; CA, carbohydrate antigen; CEA, carcinoembryonic antigen; CLE, confocal laser endomicroscopy; ERCP, endoscopic retrograde cholangiopancreatography; EUS, endoscopic ultrasound; FNA, fine-needle aspiration; HPB, hepato-pancreato-biliary; PDAC, pancreatic ductal adenocarcinoma; TE, transient elastography.
